# Study on Zinc Oxide-Based Electrolytes in Low-Temperature Solid Oxide Fuel Cells

**DOI:** 10.3390/ma11010040

**Published:** 2017-12-28

**Authors:** Chen Xia, Zheng Qiao, Chu Feng, Jung-Sik Kim, Baoyuan Wang, Bin Zhu

**Affiliations:** 1 Hubei Collaborative Innovation Center for Advanced Organic Chemical Materials, Faculty of Physics and Electronic Science, Hubei University, Wuhan 430062, China; cxia@kth.se (C.X.); qiaozheng@hgnu.edu.cn (Z.Q.); musia0803@163.com (C.F.); 2 College of Mechanical and Electrical Engineering, Huanggang Normal University, Huanggang 430062, China; 3 Department of Energy Technology, KTH Royal Institute of Technology, 10044 Stockholm, Sweden; 4 Department of Aeronautical & Automotive Engineering, Loughborough University, Loughborough LE11 3TU, UK; J.Kim@lboro.ac.uk

**Keywords:** semiconducting-ionic conductor, solid oxide fuel cells, zinc oxide, composite electrolyte, proton conduction

## Abstract

Semiconducting-ionic conductors have been recently described as excellent electrolyte membranes for low-temperature operation solid oxide fuel cells (LT-SOFCs). In the present work, two new functional materials based on zinc oxide (ZnO)—a legacy material in semiconductors but exceptionally novel to solid state ionics—are developed as membranes in SOFCs for the first time. The proposed ZnO and ZnO-LCP (La/Pr doped CeO_2_) electrolytes are respectively sandwiched between two Ni_0.8_Co_0.15_Al_0.05_Li-oxide (NCAL) electrodes to construct fuel cell devices. The assembled ZnO fuel cell demonstrates encouraging power outputs of 158–482 mW cm^−2^ and high open circuit voltages (OCVs) of 1–1.06 V at 450–550 °C, while the ZnO-LCP cell delivers significantly enhanced performance with maximum power density of 864 mW cm^−2^ and OCV of 1.07 V at 550 °C. The conductive properties of the materials are investigated. As a consequence, the ZnO electrolyte and ZnO-LCP composite exhibit extraordinary ionic conductivities of 0.09 and 0.156 S cm^−1^ at 550 °C, respectively, and the proton conductive behavior of ZnO is verified. Furthermore, performance enhancement of the ZnO-LCP cell is studied by electrochemical impedance spectroscopy (EIS), which is found to be as a result of the significantly reduced grain boundary and electrode polarization resistances. These findings indicate that ZnO is a highly promising alternative semiconducting-ionic membrane to replace the electrolyte materials for advanced LT-SOFCs, which in turn provides a new strategic pathway for the future development of electrolytes.

## 1. Introduction

In the preceding decades, fuel cells (FC) technologies have attracted enormous attention for power generation due to the imperious demand of humankind for sustainable energy resources [1,2]. As a typical category of FC technologies, solid oxide fuel cells (SOFCs) are currently receiving ever-increasing research interest because of their distinguishing advantages of high energy conversion efficiency, low greenhouse gas emissions and excellent fuel flexibility [3,4,5]. Unfortunately, current high-temperature SOFCs suffer from high manufacturing costs and technological complexities, due to the fact that yttria-stabilized zirconia (YSZ) electrolyte requires high temperatures (800–1000 °C) or precisely controlled thin film quality by advanced technologies to reach a sufficient ionic conductivity [6,7]. On the other hand, intermediate-temperature (600–800 °C) SOFCs are subject to an issue regarding the reduction reaction of Samarium-doped ceria (SDC) electrolyte in hydrogen atmosphere, which introduces additional electronic conduction and thus results in serious power loss to the cell [8]. Therefore, to realize the widespread application of SOFCs, it is highly critical to overcome the barriers of high-temperature operation and material degradation to develop advanced low-temperature (300–600 °C) SOFCs (LT-SOFCs). Since the electrolyte layer is well known as the heart of a fuel cell device in determining the operational temperatures and durability as well as the ultimate energy conversion efficiency, new strategies for excavating alternative electrolytes with high and stable ionic conductivity at reduced temperatures are strongly desired. 

To address this challenge, an efficacious approach based on semiconducting ionic conductors has been proposed very recently to replace the conventional electrolyte YSZ and SDC [9,10,11,12,13,14,15]. The developed materials have exhibited extraordinarily high ionic conductivity superior to those of YSZ and SDC, showing tremendous potential as membrane layer in LT-SOFCs [14,15]. For instance, a breakthrough study on SmNiO_3_ reported a high protonic conductivity in such perovskite semiconductor that compare favorably with those of best-performing solid electrolytes. The corresponding SOFC with Pt/SmNiO_3_/Pt geometry demonstrated dramatic power output of 225 mW cm^−2^ at 500 °C [16]. Tao et al. also demonstrated that good proton conduction (0.1 S cm^−1^ at 500 °C) can be obtained in semiconductor Li_x_Al_0.5_Co_0.5_O_2_ [17]. Our previous work also detected high ionic transport in a natural hematite (α-Fe_2_O_3_) and applied the semiconducting hematite electrolyte into SOFC, observing an impressive power density of 467 mW cm^−2^ at 600 °C [18,19]. In addition to these single phase semiconductors, high ionic conduction is also found in hetero-structured materials. Garcia-Barriocanal et al. reported a colossal ionic conduction at the interfaces of ionic conductor/semiconductor hetero-structure YSZ/SrTiO_3_, indicating that substantial ionic conductivity can be achieved even close to room temperature [20]. A series of composite materials consisting of semiconductors and ionic conductors such as Li_0.15_Ni_0.45_Zn_0.4_O_x_/SDC and SDC/Na_2_CO_3_-Sr_2_Fe_1.5_Mo_0.5_O_6−δ_ were also applied as membranes in SOFCs, revealing significantly enhanced ionic conductivity as compared to single phase ionic conductors [21,22,23,24]. A new fuel cell technology, named as electrolyte-layer free fuel cell (EFFC) or semiconductor-ion membrane fuel cell (SIMFC) designed by energy band alignment and perovskite solar cell principle has been proposed to realize better integration and functionality of these materials [11,14]. Such type of cell device is assembled using Ni_0.8_Co_0.15_Al_0.05_Li-oxide (NCAL) as electrodes into a typical configuration similar to perovskite solar cell: NCAL (ETL)/semiconducting ionic conductor (function layer)/NCAL (HTL) (ETL and HTL mean electron transport layer and hole transport layer), managing to achieve better fuel cell performances in a simpler device architecture [22].

The semiconductor ZnO has gained substantial interest in the research community and industrial applications because of its peculiar properties, such as excellent thermal stability, good oxidation resistance, considerable optoelectronic properties, and band gap in the near ultraviolet [25]. It is well known not only as a versatile semiconductor but also as a probable oxygen-ion conductor due to the enrichment of oxygen vacancies at high temperature [26]. It has been reported that the oxygen vacancy is a deep donor in ZnO with a (2+/0) transition level at ~1.0 eV below the bottom of the conduction band [27]. Liu et al. observed that addition of 0.5 wt % ZnO increased the ionic conductivity of YSZ by as much as 120% at 800 °C [28]. Furthermore, protons also may exist in ZnO and doped ZnO due to the fact that hydrogen is easily ionized to protons in oxide lattice. As reported, it is detected the concentration of protons in ZnO increases with elevating temperature [29]. Economically, ZnO is a cost-effective material in practical applications, for that it is able to be synthesized by remarkably simple crystal-growth technologies. Therefore, taking advantage of the properties of ZnO and following the above strategy, this work accesses the utility of ZnO-based materials for electrolytes in LT-SOFC. Two types of fuel cells are fabricated using pure ZnO and ZnO-LCP (La/Pr-doped CeO_2_) composite as membrane layer, respectively, sandwiched between two NCAL electrodes. The structure, morphology and electrical properties of the materials are investigated. The performances of the cells are evaluated within a low temperature range of 450–550 °C.

## 2. Experimental Section

ZnO powders were obtained through a simple pre-sintering of commercial ZnO at 650 °C for 2 h. The sintered powders were ground thoroughly for electrolyte uses and further composite preparation. LCP (La_0.33_Ce_0.6_2Pr_0.05_O_2−δ_) powder was synthesized through an 800 °C heat treatment of LaCePr-carbonates, which is a mixture of lanthanum, cerium, and praseodymium carbonates. Afterwards, ZnO-LCP composite was prepared by blending the sintered ZnO with LCP in a mass composition of 1:1. The resultant mixture was heated again at 800 °C for 2 h and ground completely to obtain the eventual homogeneous ZnO-LCP composite material. The commercial ZnO was purchased from Sigma Aldrich, Shanghai, China, and the raw material LaCcPr-carbonate was obtained from a rare-earth company in Baotou, China. Additionally, the electrode material NCAL was processed in a slurry form by mixing NCAL powder with terpineol solvent. The resultant slurry was pasted onto Ni-foam and desiccated at 120 °C for use as an electrode and current collector. The commercial NCAL was purchased from Tianjin Bamo Science and Technology Joint Stock Ltd., Tianjin, China.

Regarding the fabrication of fuel cells, two fuel cell devices were assembled based on ZnO electrolyte and ZnO-LCP composite, respectively, with two pieces of Ni-foam pasted by NCAL on both sides in each case; subsequently the three layers were pressed uniaxially under a load of 200–250 MPa into one tablet. For comparison purpose, a device based on LCP electrolyte was also fabricated in the same configuration. The resulting fuel cell devices, NCAL/ZnO/NCAL, NCAL/ZnO-LCP/NCAL, and NCAL/LCP/NCAL are roughly 2 mm in thickness and 13 mm in diameter (active area of 0.64 cm^2^). All devices, were pre-treated using an in situ heating step at 600 °C for 1 h after being mounted into the testing setup, before performance measurements at 450–550 °C.

The crystal structures of samples were studied using a Bruker D8 Advanced X-ray diffractometer (XRD, Bruker Corporation, Karlsruhe, Germany) with Cu Kα (λ = 1.54060 Å) as the source, with tube voltage at 45 kV and current of 40 mA. The particle morphology of powder samples, cross section and elemental composition of fuel cell device were investigated using a JEOL JSM7100F field emission scanning electron microscope (FE-SEM, Carl Zeiss, Oberkochen, Germany) under an accelerating voltage of 200 kV, and the equipped energy dispersive spectrometer (EDS, Carl Zeiss, Oberkochen, Germany) that operated at 15 kV. 

The electrochemical impedance spectra (EIS) of fuel cells were measured in H_2_/air atmosphere using an electrochemical work station (Gamry Reference 3000, Gamry Instruments, Warminster, PA, USA). The measurement was performed under open circuit voltage (OCV) conditions, and the applied frequency range was 0.1–10^6^ Hz with a AC signal voltage of 10 mV in amplitude. The performance measurements for fuel cells were carried out on a programmable electronic load instrument (IT8511, ITECH Electrical Co., Ltd., Nanjing, China) at 450–550 °C with humidified hydrogen as fuel (120–140 mL min^−1^) and air as the oxidant (120–150 mL min^−1^).

## 3. Results and Discussion

### 3.1. Crystalline Structure and Morphology

**Figure 1:**
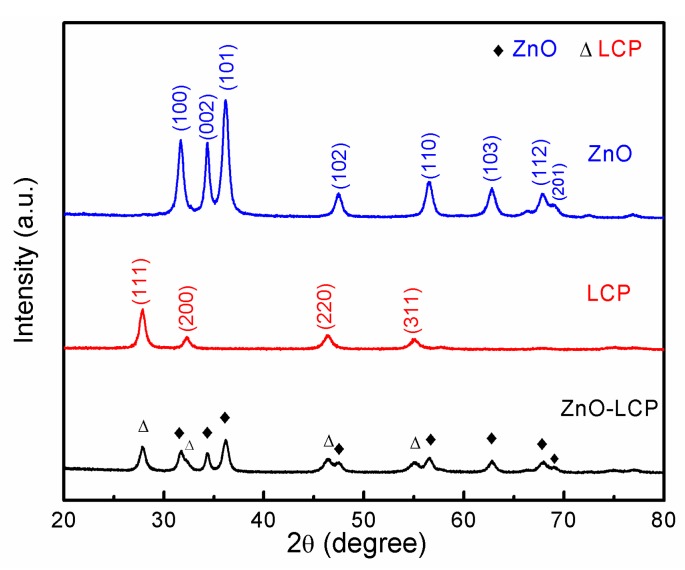
XRD patterns of the prepared ZnO, LCP and ZnO-LCP composite.

The XRD patterns of the prepared ZnO, LCP and ZnO-LCP composite are presented in Figure 1. The XRD of ZnO displays a series of characteristic diffraction peaks that correspond to the (100), (002), (101), (102), (110), (103) and (112) planes in JCPDS File No. 36-1451, which can be well indexed to the typical hexagonal wurtzite structure of zinc oxide [30]. The XRD diagram of LCP is characteristic of a cubic fluorite structure, with a slight shift to lower angle compared with the standard pattern of ceria (JCPDS File No. 34-0394), echoing the fact that LCP is a La/Pr co-doped CeO_2_ as reported previously [31]. In the diffractogram of ZnO-LCP composite, all diffraction peaks emerged can be assigned to wurtzite phase of ZnO and cubic fluorite phase of LCP, no extra phases and peak shift could be identified, which confirms that no chemical interaction occurred between the two materials. Compared to the XRD patterns of ZnO and LCP, the composite sample shows less intense peaks, revealing a larger full width at half maximum (FWHM) of the characteristic peak. According to the Scherrer equation $D=\frac{K\gamma }{\mathrm{Bcos}\theta }$, it can be calculated that the average grain size (D) of the material decreased from 26 (ZnO) and 15 (LCP) to 12 nm (ZnO-LCP). Moreover, the interplanar spacing values and lattice parameters of ZnO and LCP from the XRD analysis are given in Table 1. 

**Figure 2:**
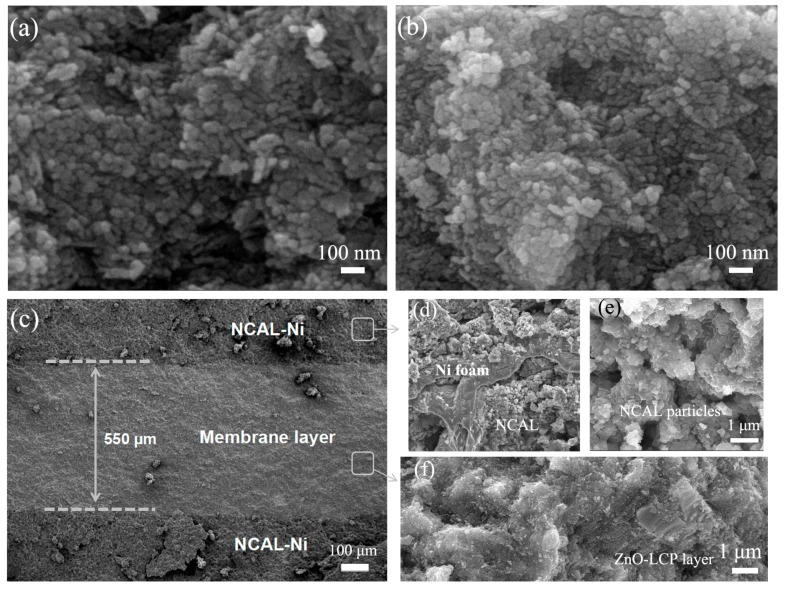
SEM images of (**a**) the resultant ZnO power and (**b**) ZnO-LCP composite; (**c**) Cross-sectional morphology of ZnO-LCP cell; (**d**) NCAL-Ni electrode; (**e**) NCAL particles and (**f**) the intermediate ZnO-LCP layer after operation.

The micro-structure of the resultant materials and fuel cell device were investigated by SEM. Figure 2a,b shows the recorded morphology of ZnO particles and ZnO-LCP particles. As can be seen, both ZnO and ZnO-LCP exhibit nano-sized particles and irregular shape particles, which is owing to the utilization of commercial and industrial-grade materials without elaborate control of nano-structure, while the composite material appears to be made up of more condensed particles with better distributions. Moreover, the average grain/particle size of ZnO-LCP were found to be smaller than that of ZnO, which is in good agreement with the XRD result. The observed size values are larger than the calculated grain size results according to the Scherrer equation, indicating that small grains aggregated in the materials and formed larger particles. 

Figure 2c illustrates a cross-sectional SEM image of the NCAL/ZnO-LCP/NCAL cell after operation, clearly displaying three individual layers consisting of a membrane layer with thickness of 550 µm and two porous NCAL-Ni electrode layers. The ZnO-LCP membrane layer adheres well with the NCAL-Ni layer without any delamination trace even after scissoring treatment for SEM characterization, as an indication of satisfactory mechanical strength of the device. Three high-magnification images of the NCAL-Ni layer, NCAL particles and the intermediate layer are further presented in Figure 2d,e, respectively. Figure 2d shows a clear view of the NCAL particles located in the three dimension woven structure of Ni-foam, forming a porous structure, which is ulteriorly confirmed in Figure 2e. The particle size of NCAL ranges from 50 to 200 nm. Additionally, as the core component of the fuel cell, ZnO-LCP layer exhibits a gas-tight structure in Figure 2f while no distinct sign of cracking can be observed. Apart from blocking gas leakage/crossover during operation, this dense layer can also ensure fast ion transport and thus aid in reducing the internal resistance of the single cell. 

**Figure 3:**
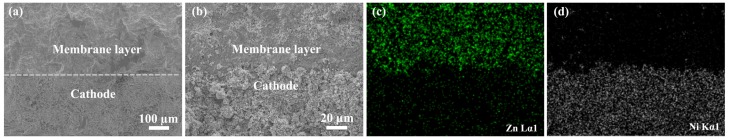

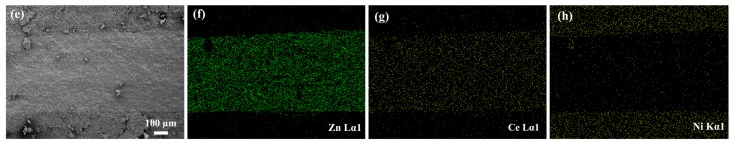
(**a**) SEM image and (**b**) detailed morphology of the membrane/cathode interface for ZnO-LCP cell after operation, and the corresponding elemental mappings for (**c**) Zn and (**d**) Ni; (**e**) Electron image of the cross-section for ZnO-LCP cell; and the corresponding elemental mappings for (**f**) Zn; (**g**) Ce and (**h**) Ni after operation.

In order to study the compatibility of the cathode and electrolyte membrane materials, Figure 3a presents a SEM image of the NCAL/ZnO-LCP/NCAL cell focusing on the membrane/cathode interface region after operation, and Figure 3b gives a detailed morphology on the basis of Figure 3a, which verifies the dense layer of ZnO-LCP membrane and porous structure of cathode again. The elemental mappings for Zn (elements only from membrane) and Ni (element only from electrode) in Figure 3c,d clearly distinguish the interface between membrane and cathode. These results indicate that ZnO-LCP membrane layers were well bonded with the NCAL cathode layer during operations without any interlaminar separation or fissure, revealing a good thermal compatibility between the membrane materials and cathode materials. Figure 3e–h further give the EDS analysis results based on the cross-section of the ZnO-LCP cell after operation. The borders between membrane layer and electrode are clear and uniform, confirmed by the elemental mappings of Zn, Ce and Ni. It reflects that there was no obvious elemental interdiffusion or segregation occurred at the interfaces during operation, which excludes the possibility of any undesired secondary reaction, and thus confirms the good chemical compatibility of the electrode and electrolyte materials.

### 3.2. Electrochemical Performance

**Figure 4:**
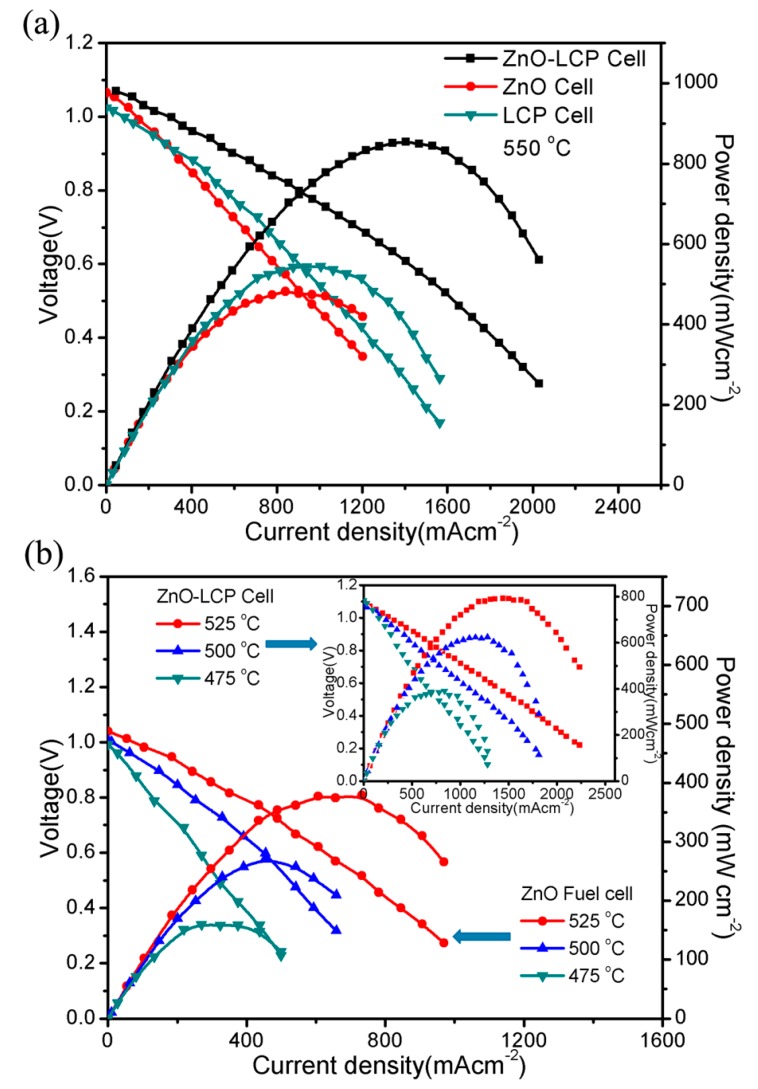
(**a**) Electrochemical performance for fuel cells based on ZnO, LCP and ZnO-LCP at 550 °C for comparison (**b**) Low-temperature performance of fuel cells with ZnO and ZnO-LCP at various temperatures.

Figure 4 shows the typical current-voltage (I-V) and current-power (I-P) characteristics for the three fuel cells based on ZnO, LCP, and ZnO-LCP electrolytes, respectively at 550 °C. From Figure 3a, it can be observed that ZnO cell delivers an OCV of 1.06 V and a maximum power density of 482 mW cm^−2^, slightly lower than that of LCP cell with 540 mW cm^−2^ in peak power density. This is the first demonstration of ZnO in fuel cell device that shows considerable performance at low temperature. It suggests the tremendous potential of ZnO from scientific and technological as well as applied perspectives for electrolyte uses. We also note that the performance is comparable to that of a newly reported thin-film SOFC based on YSZ/GDC (Gd-doped CeO_2_) bi-layer electrolyte [32], and even superior to some other SOFCs using ceria-based electrolytes [33,34]. In the case of the ZnO-LCP cell, a higher OCV of 1.07 V and significantly enhanced power density of 864 mW cm^−2^ were attained at 550 °C compared to other two cells. The power output manifested an almost 2-fold increment over that of ZnO cell. This sharp enhancement is most likely explained by the enhanced ionic conductivity in ZnO-LCP composite through interfacial conduction effect, as confirmed formerly in a number of semiconducting ionic conductors [15,19,35,36].

Based upon the above investigations, the ZnO-based cells were further assessed at reduced temperatures from 450 to 525 °C, with a 30-min dwelling time at each testing point to stabilize the cell. As shown in Figure 4b, the ZnO cell exhibits boosted power output from 158 to 380 mW cm^−2^ at 475 to 525 °C along with a mildly raised OCV from 1 to 1.03 V. Within the same temperature range, the ZnO-LCP cell demonstrates appreciable power outputs, reaching 390, 625 and 794 mW cm^−2^ at 475, 500 and 525 °C, respectively, while the OCV fluctuates in the 1.08~1.1 V window, as shown in the inset of Figure 4b. The enhancements of power density are mainly due to the thermally activated ion transport in Zno and ZnO-LCP with the rise in temperature. The achieved high OCVs can be ascribed to the excellent catalytic activity of NCAL, which has been reported as an efficient catalyst for both anode and cathode with superior triple O^2−^/H^+^/e^−^ conduction [37], and the junction effect of the device [38]. It needs to be emphasized that the junction effect is based on a Schottky junction formed between the Co/Ni alloy layer, which was originated from the anodic NCAL via reduction reaction, and the intermediate ZnO or ZnO-LCP semiconductor layer. The Schottky barrier field in the junction points from alloy layer to semiconductor layer, playing a crucial role in preventing electrons in H_2_ supply side from passing through the device [11,14]. Consequently, though there is a significant electronic conduction in the ZnO-based electrolytes, high OCVs can be still obtained by the cells. In addition, the two cell devices were further operated at lower temperature, observed is that both cells failed to reach a sufficient OCV at 450 °C, which is chiefly due to the poor catalytic activity of the NCAL electrodes at too low temperatures [39]. However, it still can be concluded from current initial results that both ZnO and ZnO-LCP composite can function well as electrolyte membrane layer in LT-SOFCs.

### 3.3. Electrical Conductivity

**Figure 5:**
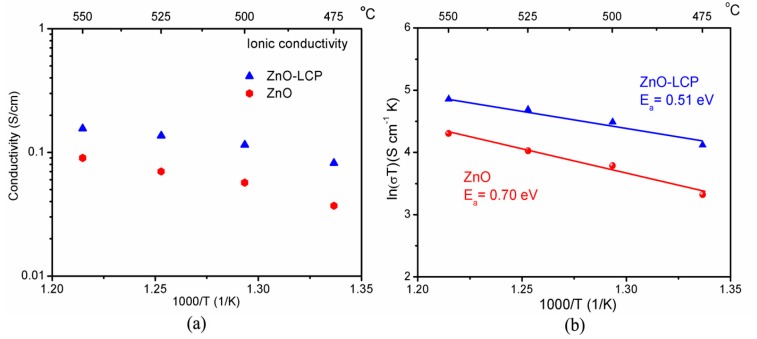
(**a**) Ionic conductivities of ZnO electrolyte and ZnO-LCP composite estimated from I-V curve result; (**b**) The corresponding activation energy of the ionic conductivities for ZnO and ZnO-LCP composite.

To understand the excellent electrochemical performances of ZnO-based fuel cells, the conductivity of the used ZnO-based materials were studied. As reported, the linear part in the central region of I-V characteristic curve mainly reflects the ohmic loss of electrolyte in a SOFC [40,41], thus ohmic resistances of the ZnO and ZnO-LCP layer can be estimated from the slope of I-V curves, from which the ionic conductivity (σ) that contributes to cell performance can be calculated according to $\sigma =\frac{L}{R\times S}$, where L is the thickness of the electrolyte layer, S denotes the effective area, and R represents the resistance. Figure 5a shows the estimated ionic conductivities for the used ZnO electrolyte and ZnO-LCP composite as a function of temperatures. The ionic conductivity of ZnO is 0.037 S cm^−1^ at 475 °C and increases to 0.09 S cm^−1^ at 550 °C. This result is slightly ahead of those of well-known oxygen ion electrolytes YSZ, GDC, Mg-doped LaGaO_3_ (LSGM), and typical proton electrolytes BaCe_0.5_Y_0.5_O_3−δ_ (BCY) and BaZr_0.1_Ce_0.7_Y_0.2_O_3−δ_ (BZCY) in previous reports [42,43]. ZnO-LCP reveals a significantly promoted ionic conductivity of 0.082 S cm^−1^ at 475 °C, which then reached as high as 0.156 S cm^−1^ at 550 °C. The corresponding activation energy (E_a_) for ionic conduction can be obtained based on Arrhenius relationship $\sigma =\frac{A}{T}\exp\;(-\frac{E_{a}}{\mathrm{kT}})$, in which T is the absolute temperature, A is a pre-exponential factor, and k represents the Boltzmann constant. As presented in Figure 5b, the E_a_ for ionic conduction of ZnO and ZnO-LCP at 475–550 °C are 0.7 and 0.51 eV, respectively, showing smaller values than those of pure O^2−^ conductors YSZ and LSGM. Particularly, the E_a_ for ZnO is close to the reported activation energies of pure proton conductors BCY and BZCY, which are in the scope of 0.66–0.78 eV [44]. Consider that protons in solid proton electrolytes generally require much lower activation energy to motivate their transport than oxygen ions, it is speculated that the ZnO-based electrolytes possess hybrid proton and oxygen ion conduction, thus resulting in a coupling lower E_a_.

**Figure 6:**
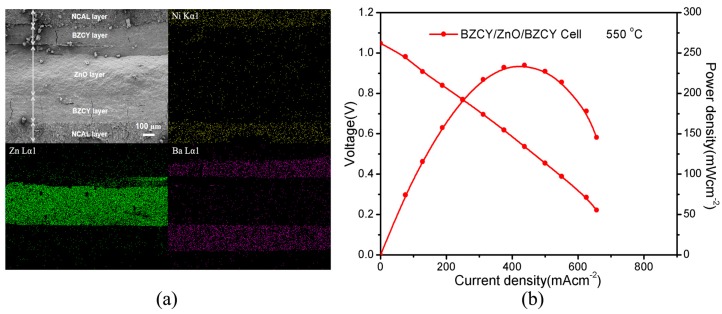
(**a**) A cross-sectional SEM image of the prepared NCAL/BZCY/ZnO/BZCY/NCAL fuel cell after operation and corresponding elemental mapping results for Ni, Zn and Ba; (**b**) Electrochemical performance of the NCAL/BZCY/ZnO/BZCY/NCAL fuel cells tested at 550 °C.

To verify the speculation, an additional experiment was undertaken to test the proton conductive behavior of ZnO electrolyte by using a O^2−^ blocking fuel cell in configuration of NCAL/BZCY/ZnO/BZCY/NCAL. Since BZCY is a state-of-the-art proton conductor with major proton conduction and negligible oxygen ion and electron conduction [45,46], the trilayer BZCY/ZnO/BZCY membrane would primarily transport protons from the anode side to cathode side while filtering out oxygen ions and electrons. By this means, the proton conductive property of ZnO can be confirmed from the performance of this multilayer cell device. This method has been reported for testing proton-related properties and conductivities of specific materials [47,48]. Figure 6a illustrates the cross-section of the device characterized by SEM after performance measurements and the corresponding elemental mappings from EDS test. As can be seen, five layers of the NCAL/BZCY/ZnO/BZCY/NCAL architecture can be clearly distinguished in SEM. This is ulteriorly identified by the elemental mappings of Ni, Zn and Ba in Figure 5a, which are exclusive from the NCAL, ZnO and BZCY layer, respectively. Few cracks are detected in the two BZCY layer membranes, probably resulting from scissoring the cross-section for SEM measurement. Figure 6b shows the cell electrochemical performance at 550 °C, exhibiting a maximum power density of 235 mW cm^−2^ with an OCV of 1.05 V. This result reflects only proton transport contribution to the power output. Therefore, from the I-V curve the proton conductivity of ZnO was calculated to be 0.05 S cm^−1^ at 550 °C. Compared to the total ionic conductivity of ZnO (0.09 S cm^−1^ at 550 °C), this value (0.05 S cm^−1^ at 550 °C) indicates that the used ZnO electrolyte might be a hybrid O^2−^/H^+^ conductor, where proton conduction dominates the total ionic conduction. However, with the multilayer configuration, the additional layers and interfaces would induce more power losses, which means the calculated value for the proton conductivity is smaller than the actual value. Therefore, it is more likely that the used ZnO electrolyte is a pure proton conductor rather than mixed proton and oxygen-ion conductor, as reported in previous study [29]. Such conductive behavior could account for the low activation energies of the materials. Our study thus confirms the proton conductive property of ZnO.

### 3.4. Impedance Spectroscopy Analysis

**Figure 7:**
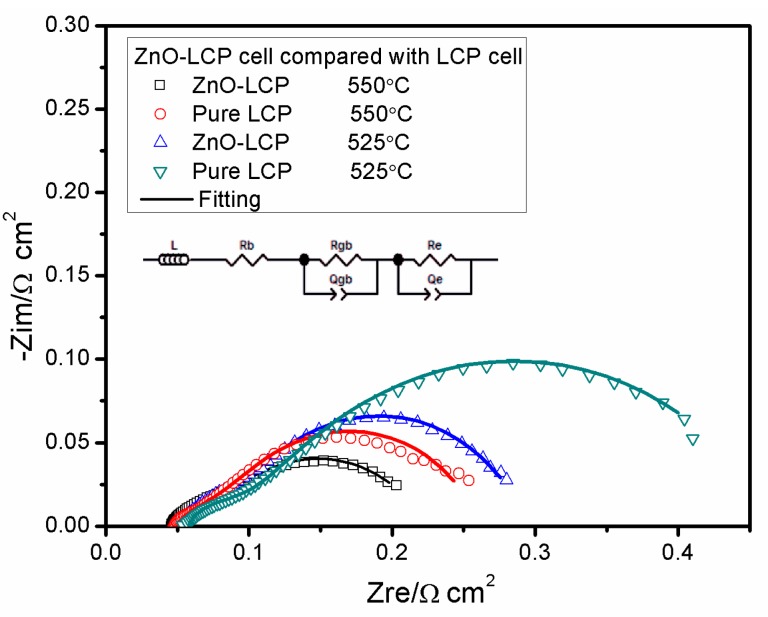
Impedance spectra for ZnO-LCP fuel cell and LCP fuel cell measured in H_2_/air at two different temperatures and the corresponding fitting lines. The inset is equivalent circuit adopted for fitting the EIS data.

Notably, the ZnO-based fuel cell exhibited significantly enhanced performance by incorporating ionic conducting LCP to form a composite. For comparative study of the electrochemical processes between our ZnO-based composite fuel cell and conventional doped-CeO_2_-based fuel cell, impedance spectroscopy analysis was carried out for the two types of cells. Figure 7 presents the EIS results of LCP cell and ZnO-LCP cell acquired in H_2_/air at 525 and 550 °C. In each impedance spectrum, the intercept with the real axis at high frequencies reflects the bulk resistance, the semicircle at intermediate frequencies represents the grain-boundary process, while the semicircle at low frequencies region corresponds to the electrode polarization behavior [41,49]. An intuitive comparison from the curves indicates that the EIS for ZnO-LCP cell have smaller high-frequency intercepts and smaller semicircles than LCP cell. We employed an empirical equivalent circuit model of LR_b_(R_gb_Q_gb_)(R_e_Q_e_) to fit the EIS data to get internal resistances information, in which L is inductance of the instrument leads and current collectors, R_b_, R_gb_ and R_e_ stand for bulk resistance, grain boundary resistance and electrode polarization resistance respectively, and Q is the constant phase element (CPE) representing a non-ideal capacitor. Thereby, R_gb_Q_gb_ and R_e_Q_e_ denote the semicircles of grain boundary conduction and electrode polarization process, respectively.

The simulated parameters extracted from the fitting results are summarized in Table 2. It can be discerned that the R_b_ of ZnO-LCP cell shows slightly smaller R_b_ than that of LCP cell at both 525 and 550 °C. With respect to the R_gb_, ZnO-LCP exhibits evidently reduced values of 0.034 Ω cm^2^ (550 °C) and 0.045 Ω cm^2^ (525 °C) as compared to LCP. This partly manifests that the migration of ions at the grain boundary is less resistive in the composite. We attributed this phenomenon to the heterophasic interfacial conduction effect at semiconductor oxide/ionic conductor oxide interface regions in hetero-structured composite material [19,50]. Such behavior has been reported in many semiconducting ionic systems, such as La_0.6_Sr_0.4_Co_0.2_Fe_0.8_O_3−δ_-Ca_0.04_Ce_0.8_Sm_0.16_O_2−δ_ (LSCF-SCDC), YSZ-SrTiO_3_, and CoFe_2_O_4_-GDC [15,20,51]. Furthermore, greater difference is observed between the R_e_ of the two devices, whereby ZnO-LCP possesses smaller R_e_ with values of 0.144 Ω cm^2^ at 550 °C and 0.197 Ω cm^2^ at 525 °C. Since these electrolyte/electrode interfacial polarization resistances often cause significant power losses in SOFCs, the smaller R_e_ of ZnO-LCP would help in attaining higher power output of the cell. The above results regarding internal resistances commendably interpret the promoted performances in ZnO-LCP composite fuel cell. 

**Figure 8:**
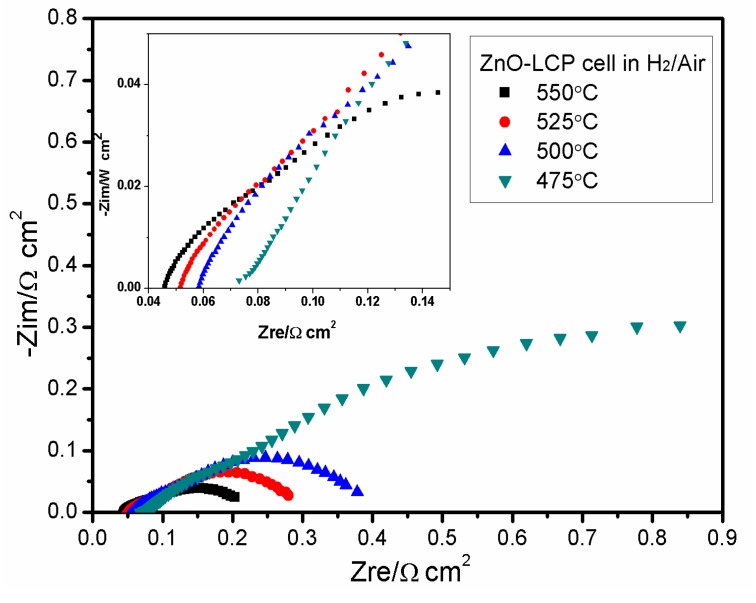
Impedance spectra of ZnO-LCP fuel cell acquired in H_2_/air at various temperatures.

Figure 8 displays the EIS of ZnO-LCP measured in H_2_/air at various temperatures. The EIS curves present in a form of flat-shaped arc or semicircle, because of the mixed electron and ion conductive behavior in the composite. With the testing temperature increases from 475 to 550 °C, the bulk resistance drops slightly from 0.072 to 0.046 Ω cm^2^, while the polarization resistance which is reflected by the intercept of arc or semicircle on the real axis reveals a dramatic shrunken tendency. For instance, at 475 °C the polarization resistance of the cell is about 1.2 Ω cm^2^ whereas the bulk resistance is smaller by one order of magnitude. It is also noted that the polarization resistance at 475 °C are far greater than those at temperatures over 500 °C. This should arise from the fact that both catalytic activity of electrode and ionic conduction of electrolyte require a sufficient thermal condition to realize their functions, suggesting that the currently designed ZnO-based cells are more applicable to operate at over 500 °C. Clearly, on the one hand, the above results prove the operational feasibility of ZnO-based electrolyte fuel cells at LT. On the other hand, it signifies that the 300–475 °C LT operation of the cell remains a huge challenge, which requires more scientific and technological studies on the materials. 

## 4. Conclusions

In summary, two zinc oxide-based electrolyte materials, pure ZnO and ZnO-LCP composite, have been developed for LT-SOFC applications for the first time. The two types of fuel cells based on pure ZnO and ZnO-LCP composite exhibited excellent power outputs of 482 and 864 mW cm^−2^ at low temperature of 550 °C, respectively. On this basis, our investigation found that ZnO electrolyte possessed decent ionic conductivity of 0.09 S cm^−1^ at 550 °C along with activation energy of 0.70 eV, while ZnO-LCP composite exhibited promoted ionic conductivity of 0.156 S cm^−1^ at the same temperature with low activation energies of 0.51 eV. These results are ahead of some standard electrolytes in previous reports. More profoundly, the proton conductive property of ZnO was detected using an oxygen-ion blocking fuel cell, showing a considerable proton conductivity of 0.05 S cm^−1^ at 550 °C. Besides, the improved performance and electrochemical processes of the ZnO-LCP cell were investigated through impedance spectra measurements. The improvements are discovered to be majorly owing to the reduced grain boundary and electrode polarization resistances. These findings suggest that zinc oxide-based semiconductors and composites are attractive materials for developing new electrolyte membranes for LT-SOFCs. It deserves more investigation into the synthesis methods and electrochemical properties regarding the electron/ion coupling effect of the materials as well as device working principle.

## Figures and Tables

**Table 1:** The lattice parameters of ZnO and LCP.

Sample	d Spacing (nm)	Lattice Constant (nm)
ZnO	0.2485 (101) plane 0.2612 (002) plane	a = b = 0.3243 c = 0.5205
LCP	0.3203 (111) plane	a = b = c = 0.5470

**Table 2:** The equivalent circuit analysis results of ZnO-LCP and LCP samples at 525 and 550 °C, the R and Q have a unit of Ω cm^2^ and S Sec^n^ cm^−2^, respectively.

Sample	T	R_b_	R_gb_	Q_gb_	n	R_e_	Q_e_	n	Chi Squared
ZnO-LCP	550 °C	0.046	0.034	0.610	0.6362	0.144	2.810	0.6307	1.675 × 10^−4^
LCP		0.048	0.042	0.820	0.506	0.173	1.650	0.7092	6.063 × 10^−4^
ZnO-LCP	525 °C	0.052	0.045	0.472	0.6312	0.197	1.325	0.7296	1.696 × 10^−4^
LCP		0.054	0.054	0.277	0.5322	0.370	1.164	0.6196	8.338 × 10^−4^
